# Range extension and natural history notes on Rocky Mountain Ridged Mussel *Gonidea angulata* (Lea, 1838) (Mollusca, Unionidae) in Canada

**DOI:** 10.3897/BDJ.14.e191607

**Published:** 2026-07-22

**Authors:** Jennifer Heron, Roxanne Jessome, Kihan Yoon-Henderson, Siena Achal

**Affiliations:** 1 British Columbia Ministry of Water, Land and Resource Stewardship, Surrey, Canada British Columbia Ministry of Water, Land and Resource Stewardship Surrey Canada; 2 British Columbia Ministry of Water, Land and Resource Stewardship, Chilliwack, Canada British Columbia Ministry of Water, Land and Resource Stewardship Chilliwack Canada; 3 British Columbia Conservation Foundation, Surrey, Canada British Columbia Conservation Foundation Surrey Canada

**Keywords:** Fraser River, freshwater mussels, Okanagan, species-at-risk

## Abstract

Rocky Mountain Ridged Mussel (*Gonidea angulata* (Lea, 1838)) is a freshwater bivalve with a global range in North America that extends from British Columbia, Canada, south to central California and east through Nevada and Idaho in the United States. In Canada, this mussel is of conservation concern and is currently assessed as endangered by the Committee on the Status of Endangered Wildlife in Canada (COSEWIC) and recommended for listing as such under the federal *Species at Risk Act* (SARA). Until recently, the species distribution in Canada was restricted to south-central British Columbia within the Okanagan watershed, with historical, yet vague records from the Kootenays, Cache Creek and Vancouver Island areas of the province. In 2023, a photograph of a Rocky Mountain Ridged Mussel shell from the confluence of the Pitt and Fraser rivers in the lower Fraser Valley of British Columbia was posted to the online citizen-science forum iNaturalist (https://www.inaturalist.ca/). The Fraser River watershed has no present-day aquatic linkages with the Okanagan watershed and the potential range extension for this mussel within a new watershed prompted targeted field surveys within the Pitt River and surrounding waterways to confirm the presence of live mussels. In 2023, 2024 and 2025, we completed surveys and confirmed a subpopulation of live Rocky Mountain Ridged Mussels within the Pitt River. This newly-confirmed subpopulation of mussels in the Fraser River watershed is of significant conservation interest, opens the door to important future research and new opportunities for monitoring and stewardship.

## Introduction

Rocky Mountain Ridged Mussel (*Gonidea angulata* (Lea 1838)) is a freshwater bivalve (Mollusca, Unionidae) first described from specimens collected in “Lewis’s River” (reported in Taylor (1981) as Snake River, Idaho, no specific locality). The species is a long-standing and undisputed taxonomic entity (Clarke 1981) and represents the only extant species in the genus (Clarke 1981, Lydeard et al. 1996, Turgeon et al. 1998). This large and distinct mussel is easily identifiable and garners attention from the public, academics and across federal, provincial, local and indigenous governments.

**Figure 1.**
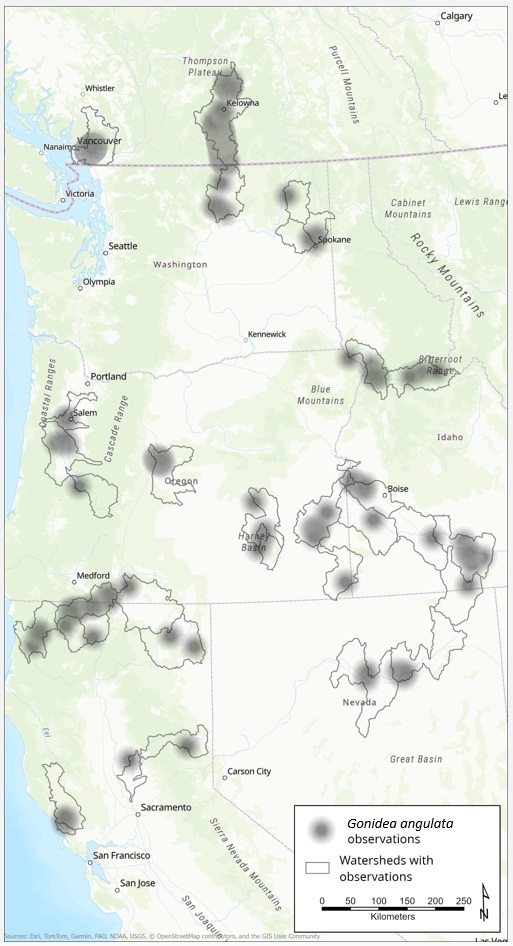
Approximate global distribution of Rocky Mountain Ridged Mussel (*Gonidea angulata*) in North America. The map highlights watersheds where the species has been recorded. This map was created using research grade data from iNaturalist (iNaturalist 2026) for the purposes of showing the mussels global distribution and does not represent a complete dataset of all known global records. Map created by Jonathan Patterson (B.C. Ministry of Water, Land and Resource Stewardship).

The global range of Rocky Mountain Ridged Mussel is restricted to western North America from southern British Columbia (B.C.), Canada, south through the western United States in Washington, Oregon to central California and east through Nevada and Idaho (Fig. 1)(Clarke 1981, COSEWIC 2010). Throughout much of its global range, the species inhabits clear, cold, freshwater rivers, larger creeks and streams with some records from larger lakes (Clarke 1981, Nedeau et al. 2009, Haag 2012, British Columbia Conservation Data Centre 2026).

**Distribution in Canada**

**Figure 2.**
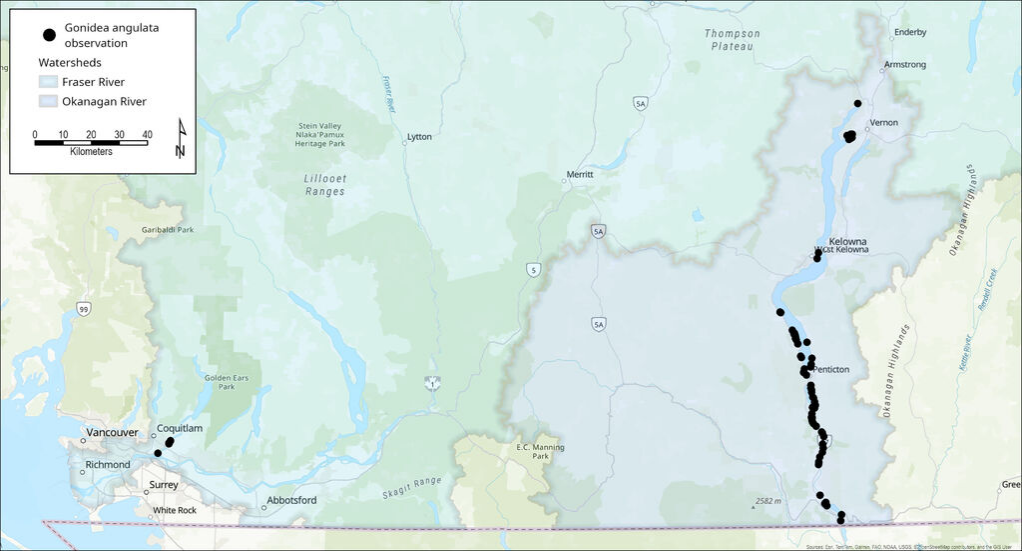
The Canadian range of Rocky Mountain Ridged Mussel (*Gonidea angulata*) is restricted to two separate watersheds in B.C.; the Okanagan River watershed and the Fraser River watershed. There are vague historical records of the mussel from Vancouver Island (1890s) and the Kootenays (unknown date), B.C., which are not shown on the map. See Table 1 for data sources. Map created by Jonathan Patterson (B.C. Ministry of Water, Land and Resource Stewardship).

An extant subpopulation of Rocky Mountain Ridged Mussel occurs within the Okanagan watershed, B.C., with records that date from 1906 to present (Fig. 2). The Okanagan watershed is approximately 8000 km^2^ and includes Okanagan Lake, Skaha Lake, Vaseux Lake and Osoyoos Lake, linked together by the Okanagan River which flows south and joins the Columbia River near Brewster, Washington, in the United States. The northernmost record of Rocky Mountain Ridged Mussel is at the north end of Okanagan Lake (Vernon) and the species is recorded from all lakes within this chain and the linkages provided by the Okanagan River. There are no documented occurrences of Rocky Mountain Ridged Mussel from the portion of the Similkameen River that is in Canada.

In 2023, a series of photographs of a Rocky Mountain Ridged Mussel shell taken at the confluence of the Pitt and Fraser rivers in the lower Fraser Valley portion of the Fraser River watershed, south-eastern B.C., Canada, was posted to the online forum iNaturalist (iNaturalist 2026). The Fraser River watershed is a separate and unconnected watershed from the Okanagan system, this location and observation being outside the previously documented Canadian range for Rocky Mountain Ridged Mussel in Canada and suggests a possible range extension. The Fraser River is the longest river and largest watershed in B.C. with an extensive network of rivers, creeks, streams and waterways that covers 220,000 km^2^ and flows from the Rocky Mountains to the Pacific Ocean at the Strait of Georgia. The area of the watershed with the photographic records of Rocky Mountain Ridged Mussel is considered the lower Fraser River and stretches from Hope west through the lower mainland and drains into the Strait of Georgia at Vancouver (Fig. 2). There are no additional historical records of the mussel from the Fraser River watershed other than shells from an archaeological site north of Cache Creek, dated from 4448 +/- 144 years before present (Lindsay 2003). There are historical, yet vague records from the Kootenays (unknown collection date) and Vancouver Island (1890s)(COSEWIC 2010, British Columbia Conservation Data Centre 2026). No additional evidence of Rocky Mountain Ridged Mussel from the Fraser River watershed, other than the iNaturalist observations, was found during our literature review.

**Morphology and Natural History**

**Figure 3.**
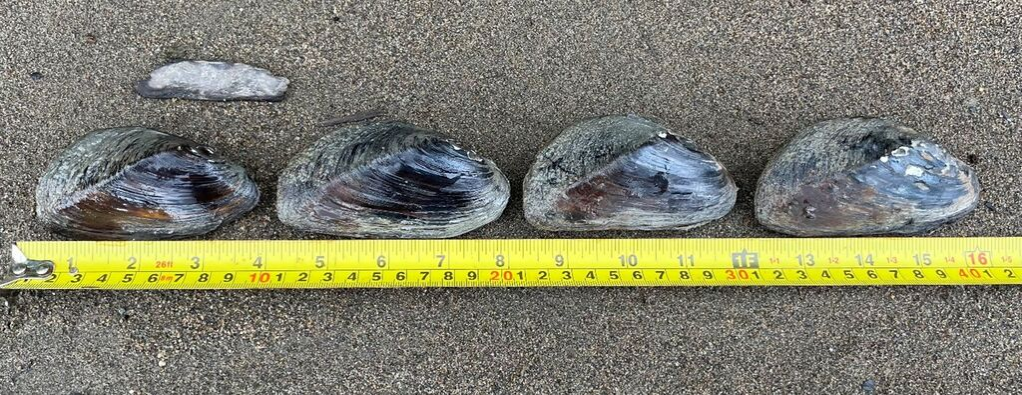
Live Rocky Mountain Ridged Mussels (*Gonidea angulata*) observed along the Pitt River, B.C., Canada on 19 September 2023. Photo Jennifer Heron (B.C. Ministry of Water, Land and Resource Stewardship).

Rocky Mountain Ridged Mussels (Fig. 3) have thick-walled trapezoidal shells with a prominent ridge that runs from the dorsal margin to the posterior edge, the main diagnostic characteristic for the species. Shell dimensions vary, however: the approximate adult size is a length of 125 mm, width 65 mm and height 40 mm (Clarke 1981). The shell colour is black-brown to yellow-brown; the inner shell surface is white or salmon, but pale blue near the edges and posterior margins. The hinge teeth (interlocking projections) are weakly developed and the two shells are connected by a ligament. The mussel is easily identifiable from both photographs and physical specimens. More information on the identification of mussels in the Pacific Northwest can be found in Clarke (1981), Nedeau et al. (2009) and Haag (2012).

The life cycle of Rocky Mountain Ridged Mussel is similar to most freshwater mussels and includes a parasitic host fish-life stage. Host fish are the main dispersal mechanism for the mussel and in Canada are suspected to include Leopard Dace (*Rhinichthys falcatus*), Longnose Dace (*Rhinichthys cataractae*), Northern Pikeminnow (*Ptychocheilus oregonensis*) and Prickly Sculpin (*Cottus asper*) (Magerøy 2015, Magerøy and Walker 2017, Snook et al. 2019). Both juvenile and adult mussels are suspension feeders and live submerged in water at or near the water/substrate interface. Mussels live in stable, low sedimentation waterways and embed within a variety of substrates including mud, silt, clay or sand, gravel and wedged between cobbles (see Nield et al. (2014) for Okanagan substrates). Detailed information on general mussel life history and habitats can be found in Haag (2012).

The objectives of our study were to confirm the presence/non-detection of a subpopulation of live Rocky Mountain Ridged Mussels within the lower Fraser River watershed and characterise their habitat, environmental variables, abundance and morphometrics. Three other native freshwater bivalves with overlapping ranges in the lower Fraser Valley closely resemble the Rocky Mountain Ridged Mussel: the Western/Oregon Floater (*Anodonta kennerlyi*/*oregonensis* clade), California/Winged Floater (*Anodonta californiensis*/*nuttalliana* clade) and Western Pearlshell (*Margaritifera falcata*). Western/Oregon Floater and California/Winged Floater are considered as clades because the taxonomy of *Anodonta* species remains uncertain, morphological differences amongst taxa are subtle and reliable identification is difficult (Chong et al. 2008, O'Brien et al. 2019, British Columbia Conservation Data Centre 2026). These mussel species were recorded to provide ecological context, assess habitat suitability and survey effectiveness. Non-native Asian Clam (*Corbicula fluminea*) was also recorded to provide additional context on habitat conditions and survey effectiveness.

## Materials and Methods

Due to the vast potential mussel habitat in the lower Fraser River watershed, we initially targeted those areas near the Pitt/Fraser River confluence where the shells from the initial iNaturalist (2026) records had been documented. Survey sites were chosen, based on ease of public access to the shoreline, safety considerations and suitable conditions for beach walk, wading and snorkel surveys. These areas are within the municipalities of Port Coquitlam, Pitt Meadows and Surrey, which all have extensive historical riparian and waterway development, interspersed amongst stretches of natural and rewilded habitat, brownfield developments, non-functional and abandoned docks and pilings. Although the land below high-tide shoreline and aquatic waterways are considered provincial government jurisdiction and open to public access, the adjacent land above the high tide is private land with limited or no access through these properties. Small watercraft (e.g. canoe, kayak) access from the river is possible although the Pitt and lower Fraser rivers are busy with larger boat and marine traffic, including recreational boat use, which increase safety concerns when completing these aquatic surveys. Site selection was further complicated by deep or fast-moving water, seasonal freshet and rising water from cumulative watershed precipitation. Under-bridge locations offered convenient and safe access to waterways, but surveys were often complicated by the presence of unhoused encampments and accumulated garbage.

In general, chosen survey sites included stretches of river protected from larger boat traffic, with slower current, extensive flat beach at low tide, shallow wading < 1 metre depth and safe from other hazards. As the Pitt and Fraser rivers are tidal, optimal survey timing is at lower tides when the muddy foreshore flats are more extensive and exposed, allowing access to the edges of the deeper main Fraser/Pitt River channels. At low tides, there are numerous deeper water pools (> 2 m depth) that remain isolated from the main river stem and these areas were targeted for ease of access and safety considerations.

Survey methods included beach-walk, wading and snorkel surveys. Beach walk surveys are a form of wandering transect and involve the surveyor(s) scanning the shoreline from the water’s edge to the high-tide area for live mussels, mussel shells and shell fragments. The surveyor typically wears a wetsuit or waders, to enable them to change course and focus on interstitial spaces, ponds, puddles, trenches and microhabitats that may conceal mussels. In some areas, the water levels change throughout the day and/or the season, exposing more shoreline and we included all these areas, where possible. We included recommendations in Mackie et al. (2008) when developing our survey methods.

Wading surveys are like beach walking and involve the surveyor(s) wearing hip-waders and wandering through the water. Wading surveys are typically at water depths less than 1.5 m and in low-flow water. At times, the surveyor uses a viewing scope to look through the water, noting mussels under the water and, if needed, reaching into the water to retrieve the mussel and confirm identification. At times, the surveyor(s) may use a rake or tool to dip into the water and retrieve a mussel. We constructed simple dipping tools by duct-taping a mesh deep-fryer basket to a broom handle, which allowed the silt and mud to wash through the mesh deep-fryer while enabling the surveyor to readily retrieve the mussel.

Snorkel surveys involve the surveyor(s) using a mask and snorkel, wearing a wetsuit and entering the water. The surveyor(s) visually scan the river bottom for live mussels and shells, hand-grubbing for mussels (i.e. sinking and moving their hands and sometimes feet to feel for mussels) or submerging to scan and hand-grub the river bottom. When a mussel was felt, the surveyor would pull the mussel above water to visually inspect and identify the mussel. The river substrates include silt, sand and mud which, when disturbed, clouded the water column and created limited visibility. Due to this limited visibility and to confirm specimen identification, mussels were removed from the substrate and brought to the surface and identification confirmed. Snorkel surveys were typically at depths less than 2.5 metres because this survey method involved the surveyor holding his/her breath and swimming to the bottom of the river, hand-grubbing to confirm mussel presence/non-detection, resurfacing for air and then repeating the survey.

During beach walk, wading and snorkel surveys, the surveyors carried a geographic positioning system (GPS) device and, where mussels were detected, the latitude and longitude was taken for each observation. When surveyors extracted a mussel from the substrate, they noted whether the mussel was prone (lying flat on the substrate) or embedded (mussel buried partially or completely, with their mantle upright in the water column) and the water depth (m) in which the mussel was located. Individual mussel measurements were taken using hand-held plastic calipers with 0.1 mm graduations. Measurements included the height (mm) which is greatest distance from the bottom/ventral to the top (dorsal) of the shell; width (mm) which is the greatest side-to-side measurement across the mussel from the valve itself; length (mm) the longest measurement of the mussel, from its anterior to posterior ends; and the total mussel wet weight (g). The substrate type was also descriptively noted.

Water quality data were recorded at each site where Rocky Mountain Ridged Mussel was observed and included the water temperature (°C), pH (how acidic or basic the water was at the collection site, measured on a scale of 0 to 14, where a pH = 7 is neutral, pH < 7 is more acidic and a pH > 7 is more basic), total dissolved solids (TDS; the total concentration of dissolved substances in water, including inorganic and organic substances, such as metals, minerals, salts and ions, measured as parts per million [ppm]) and electrical conductivity (µS [micro-siemens/cm); a measure of the saltiness of the water, freshwater is usually between 0 and 1,500 µS/cm). Data were recorded using a Hanna Combo tester model HI98129, designed for pH, EC/TDS and temperature measurements. Additional notes included the substrate (descriptive), the depth of the water where the mussel was observed (m), time of day (hh:mm) and distance mussel was recorded measured from the high-tide shoreline to its place in the river (m). When present, We also recorded Western Pearlshell and Asian Clam, both readily identifiable from their shells.

## Results

**Figure 4.**
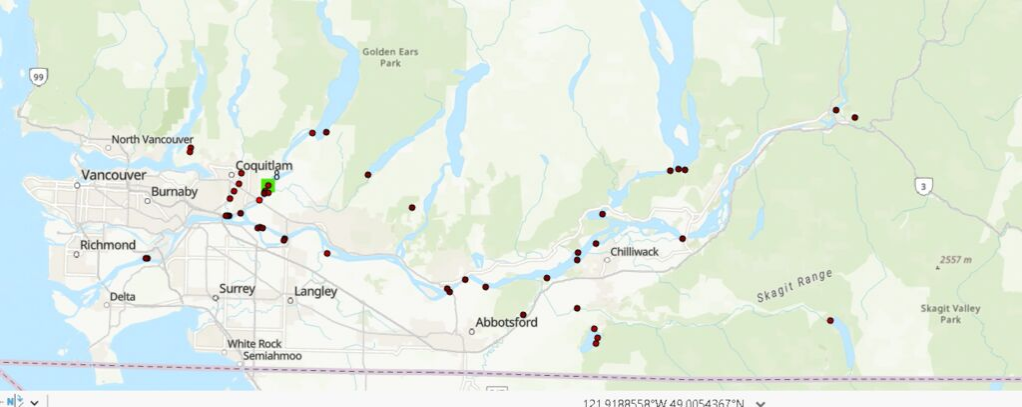
Sites surveyed for freshwater mussels in the lower Fraser River watershed in 2023–2025. The light‑green box marks the documented subpopulation of Rocky Mountain Ridged Mussel (*Gonidea angulata*) and the red circles indicate search effort with null results. Map created by Jonathan Patterson (British Columbia Ministry of Water, Land and Resource Stewardship).

**Figure 5.**
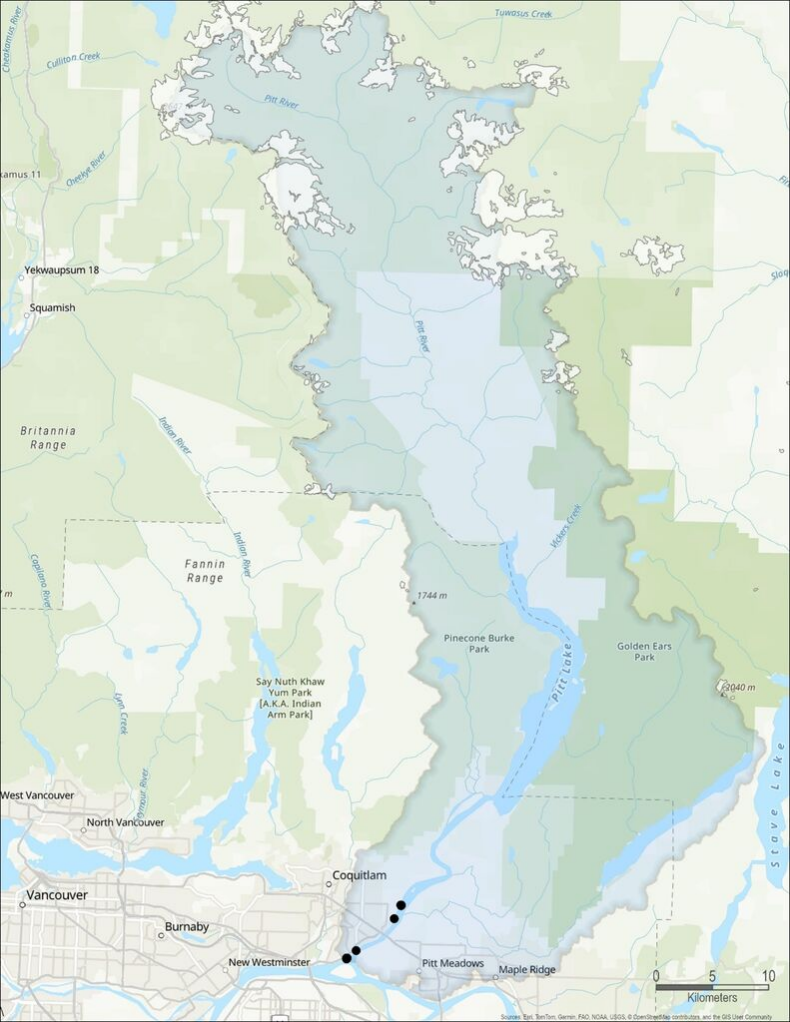
Sites where Rocky Mountain Ridged Mussel (*Gonidea angulata*) have been recorded in the Fraser River watershed, B.C., Canada. Map created by Jonathan Patterson (B.C. Ministry of Water, Land and Resource Stewardship).

Beach walking, wading and snorkel surveys were completed on various dates from September 19, 2023 to September 10, 2025. We initially conducted all three types of surveys within the same areas where the shell observations had been posted to iNaturalist (iNaturalist 2026) and we learned these sites were located on a beach adjacent to a boat-towing business. Given the chance that the shells posted to iNaturalist could have been transported from outside the Fraser River watershed (e.g., from the Okanagan) via a towed boat (rather than originating locally), and deposited in the water adjacent to the boat yard, it was important to confirm the presence of live mussels before inferring a resident Rocky Mountain Ridged Mussel subpopulation. We visited a minimum of 62 sites, with 2-9 surveyors present, and a combined minimum search effort of more than 158 hours (Fig. 4, Suppl. material 1). We documented a subpopulation of live Rocky Mountain Ridged Mussels approximately 5.4 km upstream from the site where shells had been initially posted to iNaturalist (iNaturalist 2026). A total of 81 live Rocky Mountain Ridged Mussels were recorded; four mussels on September 19, 2024; 49 on September 18, 2024 and 28 on August 8, 2025 (Fig. 5, Fig. 6a, b, c, Suppl. material 1). The subpopulation is located within a stretch of flat, mud beach that is exposed during low tides, along the west side of the lower Pitt River and adjacent to a public trail between Prairie Avenue and Dominion Avenue in Port Coquitlam.

The majority of the Rocky Mountain Ridged Mussels were found within pools that formed and became isolated from the main flow of the Pitt River when at low tide. The pools had gradual (5-10%) to steeper (10-60%) sloping edges and the depth of the pools was > 2 m, although the deepest depth of the pool was not recorded. Mussels were found both prone and embedded within substrate at water depths from 0.05 to > 2.0 m. Of the mussels found on 19 September 2024, 48 of the 49 mussels were found embedded in the muddy and fine sediments and one mussel was found prone although it was adjacent to a tidal pool edge and exposed (out of water) (Suppl. material 2). One dead specimen with its shell hinged was also recorded. Mussels were located from 54 - 74 m perpendicular distance from the high-tide of the shoreline to their place in the tidal area.

The dimensions for the 81 specimens of Rocky Mountain Ridged Mussel were recorded (Suppl. material 2). The average mussel dimensions are a length of 89.7 mm (SD 8.0 mm); width 47.3 mm (SD 4.1 mm); height 29.6 mm (SD 3.0 mm) and weight 76.9 g (SD 19.4 g). Aggregate water quality data was recorded at each site (Table 2).

Western/Oregon Floater, California/Winged Floater, Western Pearlshell and non-native Asian Clam were recorded during surveys targeting Rocky Mountain Ridged Mussel (Suppl. material 1).

## Discussion

The first records of Rocky Mountain Ridged Mussel from the lower Fraser River watershed were shells observed in 2020 and posted to iNaturalist in 2023 (iNaturalist 2026) and, following surveys in September 2023, September 2024 and August 2025, a subpopulation of live mussels has been confirmed in the Pitt River. Prior to these records, the mussel’s extant range in Canada was known only from the Okanagan watershed, with no historical records from the lower Fraser River or its tributaries, including the Pitt River. The closest records for the mussel to those in the Pitt River are within the Chehalis River in north-western Washington State (Washington Department of Fish and Wildlife 2026), for which there is no aquatic linkage with the Fraser River watershed.

In Canada, Rocky Mountain Ridged Mussel is designated as a species at risk at both the national (COSEWIC 2010, Government of Canada 2026) and provincial (British Columbia Conservation Data Centre 2026, NatureServe 2026) levels, owing to limited and fragmented distribution in the Okanagan, ongoing shoreline development and threats from the potential introduction of zebra/quagga mussels. The identification of a subpopulation in the Fraser River watershed expands the known Canadian range of Rocky Mountain Ridged Mussel and increases the number of known subpopulations; however, its hydrological isolation, unknown subpopulation size and potential distinctiveness mean that this finding does not necessarily reduce conservation concern. Determining whether the Pitt River subpopulation represents a separate designatable unit from those in the Okanagan watershed or the Chehalis River (United States) remains to be further investigated.

We consider all live Rocky Mountain Ridged Mussels found during our surveys to be adults, although there are no data that provide guidance on the distinction between juvenile and adult mussels. In general, freshwater mussels increase in size (length, width, height) as they age. The smallest mussel recorded in our survey had a length 74 mm; width 42 mm and height 31 mm (Suppl. material 2); however, the size classification for a juvenile Rocky Mountain Ridged Mussel is undetermined. The absence of detected juveniles could also reflect our inability to access suitable microhabitats (e.g. deeper and cooler parts of the Pitt River) that support juvenile burrowing.

Tidal influence in the Fraser River system presented several challenges when conducting mussel surveys; fluctuating water levels made access and detection difficult during high tide. Additionally, sediment movement and turbidity reduced the visibility and mussel detectability. We found beach walking, wading and snorkel surveys appropriate survey methods; however, survey timing was carefully planned to coincide with low tide windows. Rocky Mountain Ridged Mussel could prefer the cooler, deeper portions of the mainstem Pitt and Fraser rivers and this may partially explain our lack of detection using beach walking, wading and snorkel survey methods. This timing ensured consistent and safe access to habitats, particularly during seasonal freshet and we found that mid-August through September were ideal for survey timing. When we conducted our surveys in the Pitt River, mussels were found in deep (> 2 m) pools, within areas exposed during low-tide or at the edge of the river interface at low tide.

The aggregate water quality data from the Pitt River sites where live Rocky Mountain Ridged Mussels occur (Suppl. material 2) shows that water temperature, pH, EC and TDS are generally within tolerable ranges for freshwater mussel survival. Freshwater bivalves occur in water with near-neutral pH conditions (~ 7–8) (Cummings and Graf 2015); however, they can persist across a broader pH range of 5.6–8.3 (Fuller 1974). The average pH of 7.8 (SD 0.1) in the Pitt River is similar to the pH 7.3-8.5 recorded for Okanagan Lake (Mackie 2010); however, the available data for Okanagan Lake are not specific to Rocky Mountain Ridged Mussel habitats. The average water temperature at the sites where the mussel was detected in the Pitt River was 20.4 °C (SD 1.5°C); these measurements were at the upper end of the expected range and represent only a limited temporal window (Suppl. material 2). Without year-round temperature monitoring at these Pitt River sites, it is not possible to fully characterise seasonal variation, particularly given that Rocky Mountain Ridged Mussels occupy these habitats throughout the year. This temperature is comparable to the upper range experienced by the mussel in Okanagan Lake (Okanagan watershed), where water temperatures exceed 20 °C in summer months (Self 2024), although average temperatures are much lower (mean 9.2 °C; range 3.7 °C-22.8 °C) (District of Lake Country Utility Department 2024).

The electrical conductivity is a measure of the water’s ability to conduct electricity, which correlates with the ion concentration (e.g. salts, nutrients). The low EC of 76.3 μS/cm (SD 16.6 μS/cm) within the Pitt River system indicates clean, soft water with low mineral content, which may limit calcium availability and potentially impair shell growth and physiological functions of freshwater mussels over time (Cummings and Graf 2015). There are no data available specifically from Rocky Mountain Ridged Mussel sites in the Okanagan watershed; however, the EC in the Pitt River is slightly lower than records kept for Okanagan Lake in general, which has an average of 287 μS/cm (min. 166 μS/cm and max 343 μS/cm) (District of Lake Country Utility Department 2024). The total dissolved solids (TDS) is a measure of the combined content of all inorganic and organic substances in the water and the average for the Pitt River site is 38.2 ppm (SD 7.7), suggesting low nutrient and ion levels. Additional long-term monitoring of key water chemistry parameters across seasons and years is required within the Pitt River system before drawing further conclusions.

Given that this mussel is distinctive and readily identifiable through online citizen-science platforms, this finding presents new opportunities for community-based stewardship, public engagement and freshwater mussel conservation initiatives in B.C. Continued monitoring and collaborative efforts will be essential to improve understanding of this newly-documented subpopulation.

## Supplementary Material

01147D3A-C770-5F47-807A-D25C2C18A537 10.3897/BDJ.14.e191607.suppl1 Supplementary material 1 Search effort for freshwater bivalves in 2024 and 2025 across select waterways in the lower Fraser Valley waterways Data type null search effort, occurrences Brief description Search effort for freshwater bivalves across selected waterways in the lower Fraser Valley waterways, including location details (site name and general coordinates), survey method(s), number of surveyors, minimum surveyor search time and total cumulative search time. Species recorded during surveys included Rocky Mountain Ridged Mussel (*Gonidea angulata*), Western/ Oregon Floater (*Anodonta kennerlyi*/ *oregonensis* clade), California/Winged Floater (*Anodonta californiensis*/ *nuttalliana* clade), Western Pearlshell (*Margaritifera falcata*) and Asian Claim (*Corbicula fluminea*) File: oo_1612400.xlsx https://binary.pensoft.net/file/1612400 Jennifer Heron, Roxanne Jessome, Kihan Yoon-Henderson, Siena Achal

D34032AD-30EA-56FD-9A81-DE0604E1C6AC 10.3897/BDJ.14.e191607.suppl2 Supplementary material 2 Occurrence, abundance, shell measurements, aggregate water quality data, weight and habitat notes for specimens of Rocky Mountain Ridged Mussel (*Gonidea angulata*) recorded during surveys in the lower Fraser Valley waterways Data type occurrences and water quality Brief description The location (latitude and longitude), abundance, length (mm), width (mm), height (mm) and weight (g) of Rocky Mountain Ridged Mussel (*Gonidea angulata*) specimens and observed within the Pitt River, B.C., Canada and associated aggregate water quality data - water temperature (°C), pH, electrical conductivity (μS/cm), total dissolved solids (PPM), at sites where the mussel was recorded. File: oo_1612401.xlsx https://binary.pensoft.net/file/1612401 Jennifer Heron, Roxanne Jessome, Kihan Yoon-Henderson, Siena Achal

## Figures and Tables

**Figure 6a.**
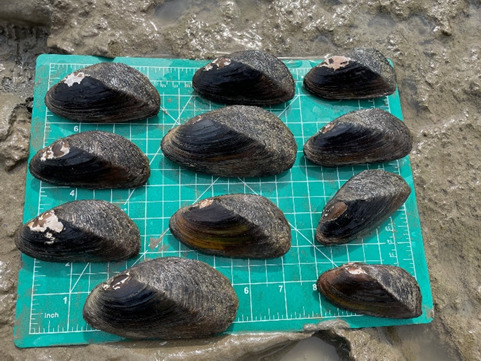
Multiple live mussels observed on 18 September 2024. All mussels were returned to their habitat. Photo Jennifer Heron (B.C. Ministry of Water, Land and Resource Stewardship);

**Figure 6b.**
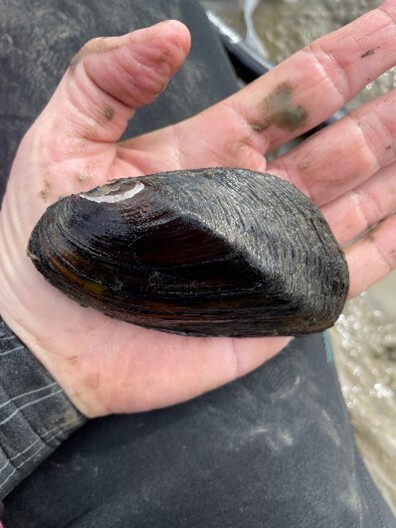
An individual live mussel observed 18 September 2024. The mussel was returned to its habitat. Photo Jennifer Heron (B.C. Ministry of Water, Land and Resource Stewardship);

**Figure 6c.**
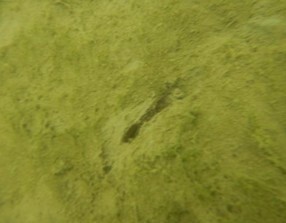
A live submerged mussel observed, embedded in the soft sediment of the Pitt River on 7 September 2023. When visibility through the Pitt River was clear, the mussels could be seen through the water with their mantle visible above the substrate. Photo Greg Wilson (B.C. Ministry of Water, Land and Resource Stewardship);

**Figure 6d.**
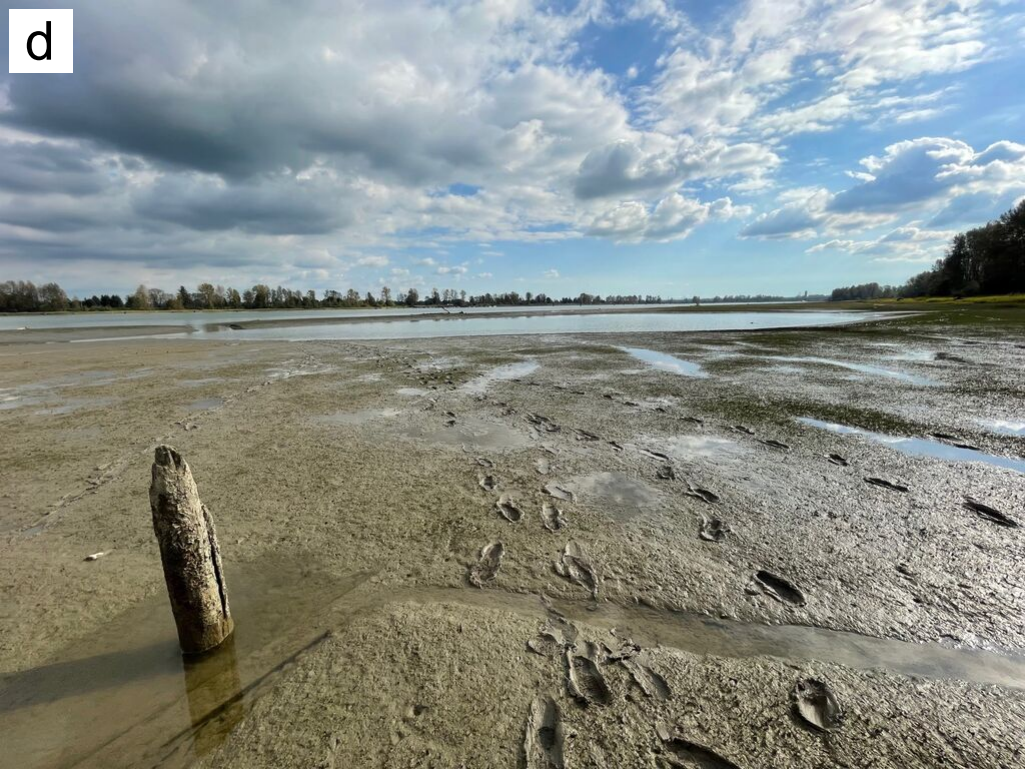
The flat expanse of beach exposed at low tide shows the soft sediment within which the mussels are found and a large pool that remains isolated from the mainstem Pitt River when the tide is low, 18 September 2024. Photo Jennifer Heron (B.C. Ministry of Water, Land and Resource Stewardship).

**Table 1.** Natural history museums, online databases and personal communications queried for records of Rocky Mountain Ridged Mussel (*Gonidea angulata*) in Canada. Data sources were queried using published synonyms for *Gonidea angulata* (Lea 1838) which include = *Anodon biangulata* G. B. Sowerby II, 1869; = *Anodon feminalis* A. Gould, 1850; = *Anodonta angulata* I. Lea, 1838; = Anodonta angulata subsp. subangulata Hemphill, 1891; = *Anodonta randalli* Trask, 1855; = Gonidea angulata subsp. haroldiana Dall, 1908; = Gonidea angulata subsp. subangulata (Hemphill, 1891); = Margarita (Anodonta) angulata (I. Lea, 1838); and = Margaron (Anodonta) angulata (I. Lea, 1838).

**Data Source**	**Specimens from the lower Fraser Valley**	**Specimens from elsewhere in BC**	**Reference**
Royal British Columbia Museum, Invertebrate Collection, Victoria, B.C.	No	Yes	Museum dataset (Choong 2025, MacIntosh 2025)
Canadian Museum of Nature Invertebrate Collection, Ottawa, Ontario	No	Yes	Canadian Museum of Nature (2026)
Beaty Biodiversity Museum at the University of British Columbia, Fish Museum Collection, Vancouver, B.C.	No	Yes	Museum dataset (Byers 2025)
British Columbia Conservation Data Centre, Species and Ecosystems Explorer	No	Yes	British Columbia Conservation Data Centre (2026)
COSEWIC (2010)	No	Yes	COSEWIC (2010)
iNaturalist (https://www.inaturalist.ca)	Yes	Yes	iNaturalist (2026) (16 observations within the lower Fraser Valley as of 6 March 2026)
Global Biodiversity Information Facility	Yes	Yes	GBIF (2026) (59 records from Canada; this collated dataset includes overlapping data from the original sources queried above)

**Table 2.** Aggregate water quality information collected from sites where Rocky Mountain Ridged Mussel (*Gonidea angulata*) was confirmed in the Pitt River, lower Fraser River watershed, B.C., Canada.

**Date**	**Location**	**Site**	**Latitude**	**Longitude**	**Water Temp**. **(°C)**	**pH**	**Electrical conduct-ivity (μS/cm)**	**Total Dissolved Solids (ppm)**
18 Sept 2024	Pitt River	Prairie Ave	49.27072	-122.71258	19.5	7.8	84	42
19 Sept 2023	Pitt River	Dominion Ave	49.262442	-122.71877	18	not recorded	13	7
8 August 2025	Pitt River	Dominion Ave	49.26425	-122.71772	22.4	7.93	72	36
Average and standard deviation (estimate based on a sample)					20.4 SD 1.5	7.8 SD 0.1	76.3 SD 16.5	38.2 SD 7.7
